# Chloroplast in Plant-Virus Interaction

**DOI:** 10.3389/fmicb.2016.01565

**Published:** 2016-10-04

**Authors:** Jinping Zhao, Xian Zhang, Yiguo Hong, Yule Liu

**Affiliations:** ^1^MOE Key Laboratory of Bioinformatics, Center for Plant Biology, Tsinghua-Peking Joint Center for Life Sciences, School of Life Sciences, Tsinghua UniversityBeijing, China; ^2^State Key Laboratory Breeding Base for Sustainable Control of Pest and Disease, Key Laboratory of Biotechnology in Plant Protection, Institute of Virology and Biotechnology, Zhejiang Academy of Agricultural SciencesHangzhou, China; ^3^Research Centre for Plant RNA Signaling, School of Life and Environmental Sciences, Hangzhou Normal UniversityHangzhou, China

**Keywords:** chloroplast, plant virus, protein interaction, virus infection, plant defense

## Abstract

In plants, the chloroplast is the organelle that conducts photosynthesis. It has been known that chloroplast is involved in virus infection of plants for approximate 70 years. Recently, the subject of chloroplast-virus interplay is getting more and more attention. In this article we discuss the different aspects of chloroplast-virus interaction into three sections: the effect of virus infection on the structure and function of chloroplast, the role of chloroplast in virus infection cycle, and the function of chloroplast in host defense against viruses. In particular, we focus on the characterization of chloroplast protein-viral protein interactions that underlie the interplay between chloroplast and virus. It can be summarized that chloroplast is a common target of plant viruses for viral pathogenesis or propagation; and conversely, chloroplast and its components also can play active roles in plant defense against viruses. Chloroplast photosynthesis-related genes/proteins (CPRGs/CPRPs) are suggested to play a central role during the complex chloroplast-virus interaction.

## Introduction

Plant viruses, as obligate biotrophic pathogens, attack a broad range of plant species utilizing host plants' cellular apparatuses for protein synthesis, genome replication and intercellular and systemic movement in order to support their propagation and proliferation. Virus infection usually causes symptoms resulting in morphological and physiological alterations of the infected plant hosts, which always incurs inferior performance such as the decreased host biomass and crop yield loss.

The most common viral symptom is leaf chlorosis, reflecting altered pigmentation and structural change of chloroplasts. Viral influence on chloroplast structures and functions usually leads to depleted photosynthetic activity. Since the first half of the twentieth century, an increasing number of reports on a broad range of plant-virus combinations have revealed that virus infection inhibits host photosynthesis, which is usually associated with viral symptoms (Kupeevicz, 1947; Owen, 1957a,b, 1958; Hall and Loomis, 1972; Mandahar and Garg, 1972; Reinero and Beachy, 1989; Balachandran et al., 1994b; Herbers et al., 2000; Rahoutei et al., 2000; Guo et al., 2005; Christov et al., 2007; Kyseláková et al., 2011). It is suggested that modification of photosynthesis is a common and conserved strategy for virus pathogenesis to facilitate infection and to establish an optimal niche (Gunasinghe and Berger, 1991). The disturbance of chloroplast components and functions may be responsible for the production of chlorosis symptoms that are associated with virus infection (Manfre et al., 2011).

A series of typical changes followed by chlorotic symptoms imply the occurrence of chloroplast-virus interactions. These changes include (1) fluctuation of chlorophyll fluorescence and reduced chlorophyll pigmentation (Balachandran et al., 1994a), (2) inhibited photosystem efficiency (Lehto et al., 2003), (3) imbalanced accumulation of photoassimilates (Lucas et al., 1993; Olesinski et al., 1995, 1996; Almon et al., 1997), (4) changes in chloroplast structures and functions (Bhat et al., 2013; Otulak et al., 2015), and (5) repressed expression of nuclear-encoded chloroplast and photosynthesis-related genes (CPRGs) (Dardick, 2007; Mochizuki et al., 2014a), (6) direct binding of viral components with chloroplast factors (Shi et al., 2007; Bhat et al., 2013; Zhao et al., 2013).

In fact, the chloroplast itself is a chimera of components of various origins coming from its bacterial ancestors, viruses and host plants. For example, chloroplast contains the nuclear-encoded phage T3/T7-like RNA polymerase (Hedtke et al., 1997; Kobayashi et al., 2001; Filée and Forterre, 2005). It is not surprising that chloroplast has an important role in plant-virus interactions. Indeed, more and more chloroplast factors have been identified to interact with viral components (Table 1). These factors are involved in virus replication, movement, symptoms or plant defense, suggesting that viruses have evolved to interact with chloroplast.

**Table 1:** **Chloroplast factors interacting with virus nucleic acids or proteins**.

Plant Virus^*^	**Virus components**	**Chloroplast factors**	**Subcellular localization**	**Biological process**	**References**
**ssRNA POSITIVE-STRAND VIRUSES**					
***Potexvirus*****/*****Alphaflexiviridae***					
Alternanthera mosaic virus (AltMV)	TGB3	Chloroplast membrane	Chloroplast	Cell-to-cell movement, long-distance movement, symptom	Lim et al., 2010
		PsbO	Surrounding chloroplast	Symptom	Jang et al., 2013
Bamboo mosaic virus (BaMV)	RNA 3′ UTR	cPGK	Chloroplast Cytoplasm,	Replication	Cheng et al., 2013
Potato virus X (PVX)	CP	Plastocyanin	Chloroplast	Symptom	Qiao et al., 2009
***Alfamovirus*****/*****Bromoviridae***					
Alfalfa mosaic virus (AMV)	CP	PsbP	Cytoplasm	Replication	Balasubramaniam et al., 2014
***Cucumovirus*****/*****Bromoviridae***					
Cucumber mosaic virus (CMV)	1a, 2a	Tsip1	Cytoplasm	Replication	Huh et al., 2011
Cucumber mosaic virus Y strain satellite RNA (CMV-Y-sat)	22-nt vsiRNA^**^	*ChlI mRNA*	Cytoplasm	Symptom	Shimura et al., 2011; Smith et al., 2011
***Potyvirus*****/*****Potyviridae***					
Potato virus Y (PVY)	CP	RbCL	–	Symptom	Feki et al., 2005
	HC-Pro	MinD	Cytoplasm	Symptom	Jin et al., 2007
		CF1β	Chloroplast	Symptom	
Onion yellow dwarf virus (OYDV)	P3	RbCL, RbCS	–	–	Lin et al., 2011
Plum pox virus (PPV)	CI	PsaK	–	Host defense	Jimenez et al., 2006
Sugarcane mosaic virus (SCMV)	HC-Pro	Fd V	Cytoplasm	Symptom	Cheng et al., 2008
Soybean mosaic virus (SMV)	P1	Rieske Fe/S	–	Symptom	Shi et al., 2007
	P3	RbCL, RbCS	–	–	Lin et al., 2011
Shallot yellow stripe virus (SYSV)	P3	RbCL, RbCS	–	–	Lin et al., 2011
Turnip mosaic virus (TuMV)	CP	37-kD protein	–	–	McClintock et al., 1998
	P3	RbCL, RbCS	–	–	Lin et al., 2011
Tobacco vein-mottling virus (TVMV)	CI	PsaK	–	Host defense	Jimenez et al., 2006
***Dianthovirus*****/*****Tombusviridae***					
Red clover necrotic mosaic virus (RCNMV)	MP	GAPDH-A	Chloroplast, Endoplasmic reticulum	Cell-to-cell movement	Kaido et al., 2014
***Pomovirus*****/*****Virgaviridae***					
Potato mop-top virus (PMTV)	TGB2	Chloroplast lipid	Chloroplast	Replication	Cowan et al., 2012
***Tobamovirus*****/*****Virgaviridae***					
Tobacco mosaic virus (TMV)	126 K replicase	PsbO	–	Host defense	Abbink et al., 2002
		NRIP	Cytoplasm, Nucleus	Host defense	Caplan et al., 2008
	126 K/183 K replicase	AtpC	VRCs	Host defense	Bhat et al., 2013
		RCA	VRCs		Host defense
	MP	RbCS	Cytoplasm	Cell-to-cell movement	Zhao et al., 2013
Tomato mosaic virus (ToMV)	CP	Fd I	Cytoplasm	Symptom	Sun et al., 2013; Ma et al., 2008
		IP-L	Thylakoid membrane	Long distance movement	Li et al., 2005; Zhang et al., 2008
	MP	RbCS	Cytoplasm	Cell-to-cell movement	Zhao et al., 2013
**ssRNA NEGATIVE SENSE VIRUSES**					
***Tenuivirus***/**Unassigned**					
Rice stripe virus (RSV)	SP	PsbP	Cytoplasm	Symptom	Kong et al., 2014
**ssDNA VIRUSES**					
***Begomovirus*****/*****Geminiviridae***					
Abutilon mosaic virus (AbMV)	MP	cpHSC70-1	Cell periphery, Chloroplast	Cell-to-cell movement	Krenz et al., 2010, 2012
**dsDNA VIRUSES**					
***Caulimovirus*****/*****Caulimoviridae***					
Cauliflower mosaic virus (CaMV)	P6	CHUP1	VRCs	Cell-to-cell movement	Angel et al., 2013

* Virus taxonomy is in format of Genus/Family.

** Virus-derived small interfering RNA.

– Not addressed. ssRNA, single-stranded RNA; ssDNA, single-stranded DNA.

In this review, we focus on the topic of how chloroplast factors and viral components interact with each other and how these interactions contribute to viral pathogenesis and symptom development, especially in virus-susceptible hosts.

## Chloroplast is involved in viral symptom production

Although the development of viral symptoms can be traced back to different causes, the disruption of normal chloroplast function has been suggested to cause typical photosynthesis-related symptoms, such as chlorosis and mosaic (Rahoutei et al., 2000). Chloroplast has been implicated as a common target of plant viruses for a long time. For instance, the severe chlorosis on systemic leaves infected by CMV in *Nicotiana tabacum* cv. Xanthi nc is associated with size-reduced chloroplasts containing fewer grana (Roberts and Wood, 1982). A second example shows that the leaf mosaic pattern caused by virus infection can be due to the layout of clustered mesophyll cells in which chloroplasts were damaged to various degrees (Almási et al., 2001). A third example shows that symptom caused by PVY infection is often associated with decrease in the number and size of host plant chloroplasts as well as inhibited photosynthesis (Pompe-Novak et al., 2001). Based on the current studies, the ultrastructural alteration of chloroplast and the reduced abundance of proteins involved in photosynthesis are the two main causes of virus induced chloroplast symptomatology (see below).

### Effect of virus infection on chloroplast structure

**Figure 1:**
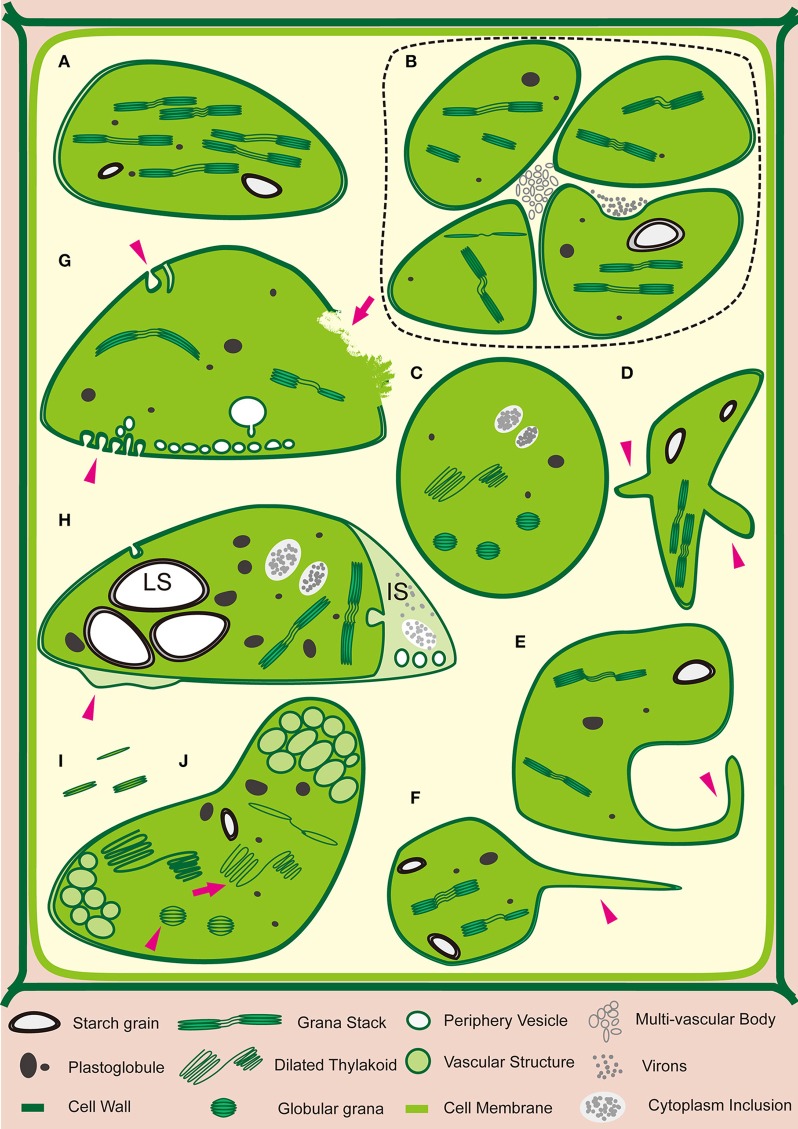
**Changes in the Ultrastructure of Chloroplasts Induced by Virus Infection. (A)** Normal chloroplast. **(B)** Aggregated chloroplasts (surrounded with dotted line). **(C)** Swollen chloroplast. **(D)** Chloroplast with membrane-bound extrusions. Arrow heads indicate membrane extrusions. **(E)** Amoeboid-shaped chloroplast, arrow head indicates chloroplast membrane extrusions. **(F)** Chloroplast with stromule, arrow head indicates the stromule. **(G)** Chloroplast with irregular out-membrane structures such as peripheral vesicle, cytoplasmic invagination, membrane proliferations and broken envelope. Arrow heads indicates cytoplasmic invaginations, arrow indicates broken envelope of chloroplast. **(H)** Chloroplast with abnormal content changes such as small vesicles, membrane proliferations (arrow head) and inter-membranous sac (IS), large starch grain (LS) and exaggeration of plastoglobules. **(I)** Disorganized grana scattering into the cytoplasm. **(J)** Chloroplast with unusual photosynthetic structures such as dilated thylakoid (arrow) and globular grana (arrow head) and vascular structures.

Successions of analysis on the ultrastructural organization of plant cells infected with viruses have been performed with electron microscopy since the 1940s. There is a stunning convergence among different host-virus systems where significant alteration or rearrangement of the chloroplast ultrastructure is correlated with the symptom development (Bald, 1948; Arnott et al., 1969; Ushiyama and Matthews, 1970; Allen, 1972; Liu and Boyle, 1972; Mohamed, 1973; Moline, 1973; Appiano et al., 1978; Tomlinson and Webb, 1978; Schuchalter-Eicke and Jeske, 1983; Bassi et al., 1985; Choi, 1996; Mahgoub et al., 1997; Xu and Feng, 1998; Musetti et al., 2002; Zechmann et al., 2003; Guo et al., 2004; El Fattah et al., 2005; Schnablová et al., 2005; Li et al., 2006; Yan et al., 2008; Laliberté and Sanfaçon, 2010; Montasser and Al-Ajmy, 2015; Zarzyńska-Nowak et al., 2015; Zhao et al., 2016). The chloroplast malformations include (1) overall decrease of chloroplast numbers and chloroplast clustering; (2) atypical appearance of chloroplast, such as swollen or globule chloroplast, chloroplast with membrane-bound extrusions or amoeboid-shaped chloroplast, generation of stromule (a type of dynamic tubular extensions from chloroplast); (3) irregular out-membrane structures such as peripheral vesicle, cytoplasmic invagination, membrane proliferations and broken envelope; (4) changes of content inside the chloroplast such as small vesicles or vacuoles in stroma, large inter-membranous sac, numerous, and/or enlarged starch grains, increase in the number and size of electron-dense granules/plastoglobules/bodies; (5) unusual photosynthetic structures such as disappearance of grana stacks, distorted, loosen, or dilated thylakoid and the disappearance of stroma; and (6) completely destroyed chloroplasts and disorganized grana scattering into the cytoplasm. In these studies, the viruses are from 12 families and have either sense ssRNA, antisense ssRNA or ssDNA genomes, covering the majority of genera and including those responsible for devastating disease. This implies that chloroplast abnormality is a common event across diverse plant-virus interactions. The types of chloroplast abnormalities caused by virus infection are summarized in Table 2 and schemed in Figure 1.

**Table 2:** **Structural changes of chloroplasts induced by virus infection**.

Plant Virus^*^	**Chloroplast Abnormality**	**Plant Host**	**Virus Factor**	**References**
**ssRNA POSITIVE-STRAND VIRUSES**				
***Potexvirus*****/*****Alphaflexiviridae***				
Potato virus X (PVX)	Invaginations of cytoplasm into chloroplast	*Datura stramonium, Solanum tuberosum*	Virus particle, Virus inclusion	Kozar and Sheludko, 1969
	Dilated granal lamella, enlarged stromal areas, thylakoid vesicles	*Nicotiana benthamiana*	CP	Qiao et al., 2009
Alternanthera mosaic virus (AltMV)	Vesicular invaginations	*Nicotiana benthamiana*	Viral RNA, TGB3	Lim et al., 2010
***Carlavirus*****/*****Betaflexiviridae***				
*Potato virus S* (PVS)	Cytoplasm invagination	*Chenopodium quinoa*	Virion	Garg and Hegde, 2000
***Cucumovirus/Bromoviridae***				
Cucumber mosaic virus isolate 16 (CMV-16)	Reduction in chloroplast number and size, completely destroyed chloroplasts and disorganized grana scattering into the cytoplasm	*Lycopersicon esculentum*	–	Montasser and Al-Ajmy, 2015
CMV P6 strain (CMV-P6)	Tiny chloroplast with fewer grana, myelin-like chloroplast-related structures	*Nicotiana tabacum*	–	Roberts and Wood, 1982
CMV Malaysian isolate	Disorganized thylakoid system, crystallization of phytoferritin macro molecules and, large starch grains	*Catharanthus roseus*	–	Mazidah et al., 2012
CMV pepo strain with CP129 substitutions	Few thylakoid membranes, no granum stacks, abnormal-shaped and hyper-accumulated starch grains	*Nicotiana tabacum*	–	Mochizuki and Ohki, 2011
CMV pepo strain VSR deficient mutant with CP129 substitutions	Fewer thylakoid membranes and granum stacks	*Nicotiana tabacum*	–	Mochizuki et al., 2014b
***Polerovirus*****/*****Luteoviridae***				
Beet western yellows virus (BWYV)	Disappearance of grana stacks, stroma lamellae, large starch grains, osmiophilic granules	*Lactuca sativa, Claytonia perfoliata*	–	Tomlinson and Webb, 1978
Sugarcane Yellow Leaf Virus (ScYLV)	Swollen chloroplast, rectangular grana stacks, more plastoglobules	*Saccharum spec.*	–	Yan et al., 2008
***Potyvirus*****/*****Potyviridae***				
Bean yellow mosaic virus (BYMV)	Increased stromal area, swollen chloroplast, loss of envelopes, dilated thylakoids, decreased chloroplast number	*Vicia faba*	–	Radwan et al., 2008
Maize dwarf mosaic virus strain A (MDMV-A)	Small vesicles, deformation of membranes, reduction in grana stack height, disappearance of osmiophilic globules, degeneration of structures	*Sorghum bicolor*	–	Choi, 1996
MDMV Shandong isolate (MDMV-SD)	Thylakoid swelling, envelope broking	*Zea mays*	–	Guo et al., 2004
Plum pox virus (PPV)	Dilated thylakoid, increase in the number and size of plastoglobuli, decreased amount of starch in chloroplasts from palisade parenchyma	*Prunus persica* L.	–	Hernández et al., 2006
	Dilated thylakoids, increased number of plastoglobuli, peculiar membrane configurations	*Pisum sativum*	–	Díaz-Vivancos et al., 2008
	Lower amount of starch granules, disorganized grana structure	*Prunus persica* L.	–	Clemente-Moreno et al., 2013
Potato virus Y (PVY)	Reduced chloroplast number, smaller chloroplasts with exvaginations	*Solarium tuberosum*	–	Pompe-Novak et al., 2001
	Decrease of volume density of starch, increase of volume density of plastoglobuli	*Nicotiana tabacum*	–	Schnablová et al., 2005
Sugarcane mosaic virus (SCMV)	Swollen chloroplast, increased number of plastoglobuli	*Sorghum bicolor*	–	El Fattah et al., 2005
Turnip mosaic Virus (TuMV)	Chloroplast aggregation, irregular shaped chloroplast, large osmiophilic granules, poorly developed lamellar system, few or no starch grains,	*Chenopodium quinoa*	Virus particle	Kitajima and Costa, 1973
Zucchini yellow mosaic virus (ZYMV)	Decrease of chloroplasts amount, decreased thylakoids, increased plasto-globule and starch grain in chloroplast	*Cucurbita pepo*	–	Zechmann et al., 2003
***Fijivirus*****/*****Reoviridae***				
Maize rough dwarf virus (MRDV)	Membrane disappearance, swollen grana discs, periphery vesicles	*Zea mays*	Virus particle	Gerola and Bassi, 1966
	Distorted grana and paired membranes.	*Chenopodium quinoa*	Virus particle	Martelli and Russo, 1973
***Fabavirus*****/*****Secoviridae***				
Broad bean wilt virus 2 (BBWV-2) isolate B935	Inhibited lamellar development, membrane vesiculation	*Vicia faba*	–	Li et al., 2006
BBWV-2 isolate PV131	Chloroplast with swollen or disintegrated membrane	*Vicia faba*	–	
***Tombusvirus*****/*****Tombusviridae***				
Artichoke mottled crinkle virus (AMCV)	Distorted grana and paired membranes.	*Chenopodium quinoa*	Virus particle	Martelli and Russo, 1973
Tomato bushy stunt virus (TBSV)	Large plastidial vacuole, disorganized lamellar system, multivesicular bodies originate from chloroplasts, chloroplasts clustered around a group of multivesicular bodies	*Gomphrena globosa*	Virus particle	Appiano et al., 1978
	Large inter-membranous sac, rearrangement of the thylakoids	*Datura stramonium*	–	Bassi et al., 1985
**Unassigned/*****Tombusviridae***				
Maize necrotic streak virus (MNeSV)	Chloroplast swollen, out membrane invagination	*Zea mays*	–	De Stradis et al., 2005
***Tymovirus*****/*****Tymoviridae***				
Melon rugose mosaic virus (MRMV)	Peripheral vesicles, tendency to aggregate	*Cucumis melo*	–	Mahgoub et al., 1997
Turnip yellow mosaic virus (TYMV)	Peripheral vesicles, reduction of grana number, chlorophyll content; increases in amounts of phytoferritin and numbers of osmiophilic globules	*Brassica rapa*	Viron, Viral RNA	Ushiyama and Matthews, 1970; Hatta and Matthews, 1974
Belladonna mottle virus physalis mottle strain (BeMV-PMV)	Vesicles develop in chloroplasts, vesiculations of the outer membranes	*Datura stramonium*	Viron	Moline, 1973
Wild cucumber mosaic virus (WCMV)	Double membrane vesicles in chloroplasts, single membrane vesicles surrounding chloroplasts	*Marah oreganus*	Virus particle	Allen, 1972
***Hordeivirus*****/*****Virgaviridae***				
Barley stripe mosaic virus (BSMV)	Surrounded chloroplasts, cytoplasmic invaginations into chloroplasts, aggregated chloroplasts, rearrangement of the thylakoids, electron transparent vacuoles in stroma	*Hordeum vulgare*	Viron	Carroll, 1970; Zarzyńska-Nowak et al., 2015
	Peripheral vesicles; Type1: elongated grana or anastomosed lamellae, composed of pellucid stroma, twisted or convoluted membranes forming tubular networks; Type2: swollen and contained disarranged internal membranes; Type3: electron dense stroma, cytoplasmic invaginations.	*Datura stramonium*	Genomic ssRNA	McMullen et al., 1978
	Rounded and clustered chloroplasts, cytoplasmic invaginations and inclusions at the periphery	*Nicotiana benthamiana*	TGB2, CP, γb, virus-like particle	Torrance et al., 2006
***Pomovirus/Virgaviridae***				
Potato mop-top virus (PMTV)	Large starch grains, large cytoplasmic inclusion, terminal extension,	*Nicotianabenthamiana*	Genomic RNA, CP, TGB2	Cowan et al., 2012
***Tobamovirus/Virgaviridae***				
Ribgrass mosaic virus (RMV)	Disappearance of stroma, decrease in grana lamella, Large starch grains, osmiophilic granules	*Nicotiana tabacum*	–	Xu and Feng, 1998
Tobacco mosaic virus (TMV)	Aggregates and vecuoles in chloroplast	*Lycopersicon esculentum*		Shalla, 1964
	Enlarged plastids, supergranal thylakoids, large accumulations of osmiophilic bodies	*Lycopersicon esculentum*	–	Arnott et al., 1969
	Disappearance of stroma, decrease in grana lamella, large starch grains, osmiophilic granules	*Nicotiana tabacum*	CP	Xu and Feng, 1998
	Swelling, more osmophilic plastoglobuli, loosened thylakoid structure	*Capsicuum anuum*	–	Mel'nichuk et al., 2002
TMV U5 strain	Peripheral vesicles	*Nicotiana tabacum*	Virus particle	Betto et al., 1972
TMV yellow strain	Filled with osmiophilic globules, rearranged, swollen or eliminated lamellar system, extensive chloroplast degradation	*Solanum tuberosum*	–	Liu and Boyle, 1972
TMV *flavum* strain (TMV-Flavum)	Swollen or globular chloroplast, distorted thylakoid membranes, grana depletion, unidentified granular matter	*Nicotiana tabacum*	MP, CP	Lehto et al., 2003
Tomato mosaic Virus (ToMV)	Slightly swollen and distorted cholroplast, large starch grains	*Nicotiana tabacum*	Virus particle	Ohnishi et al., 2009
ToMV L11Y strain (ToMV-L11Y)	Flaccid chloroplast, reduced thylakoid stacks and enlarged spaces between the stacks, cytoplasm penetrates into chloroplast, tubular complexes	*Nicotiana tabacum*	–	Ohnishi et al., 2009
**ssRNA NEGATIVE STRAND VIRUSES**				
***Tospovirus/Bunyaviridae***				
Tomato spotted wilt virus (TSWV)	Peripheral vesicles	*Nicotiana tabacum*	–	Mohamed, 1973
***Tenuivirus/*****Unassigned**				
Rice stripe virus (RSV)	Reduced sheets of grana stacks, increased amount and size of starch granules	*Oryza Sativa*	Virus particle	Zhao et al., 2016
	Membrane proliferations	*Nicotiana benthamiana*	NSvc4	
**ssDNA VIRUSES**				
***Begomovirus/Geminiviridae***				
Abutilon Mosaic Virus (AbMV)	Disorganization of thylakoid system, grana-stroma elimination	*Abutilon spec*	–	Schuchalter-Eicke and Jeske, 1983
	Degenerated thylakoids, more plastoglobuli, less starch, and accumulation of amorphous electron-dense material	*Abutilon selovianum*	Genomic DNA	Gröning et al., 1987
	Generation of stromules	*Nicotiana benthamiana*	MP	Krenz et al., 2012

* Virus taxonomy is in format of Genus/Family.

– Not addressed. ssRNA, single-stranded RNA; ssDNA, single-stranded DNA.

### Viral effectors are related to the chloroplast structural changes

Recent reports have revealed that viral factors, especially coat proteins (CPs), affect chloroplast ultrastructure and symptom development (see below).

Viral coat proteins (CPs) have been demonstrated as determinants of symptom phenotypes for a much long period (Heaton et al., 1991; Neeleman et al., 1991). The earlier research showed that virion-like particles or virus inclusion in chloroplast are positively related to the development of mosaic symptom caused by TMV (Bald, 1948; Shalla, 1964). The more virion-like particles accumulated in chloroplast, the more severe morphological defects of chloroplast structure occurred (Matsushita, 1965; Shalla, 1968; Granett and Shalla, 1970; Betto et al., 1972). Later researches indicate that virion-like particles in chloroplast are pseudovirions, in which chloroplast transcripts are encapsidated by TMV CPs (Shalla et al., 1975; Rochon and Siegel, 1984; Atreya and Siegel, 1989), highlighting the involvement of CPs in the alteration of chloroplast ultrastructure. TMV CP does not possess a classical chloroplast transit peptide (TP) but can be imported into chloroplast effectively in a ATP-independent mode (Banerjee and Zaitlin, 1992). The majority of TMV CPs in chloroplasts are associated with the thylakoid membranes in systemically invaded *N. tabacum* leaves (Reinero and Beachy, 1986; Hodgson et al., 1989). Various natural TMV mutants, whose CPs excessively accumulate in chloroplast, always induce more severe symptoms and aggravated inhibition of the PS II activity (Regenmortel and Fraenkel-Conrat, 1986; Reinero and Beachy, 1986, 1989; Banerjee et al., 1995; Lehto et al., 2003), suggesting that chloroplast-targeted CPs act as the inducer of chloroplast ultrastructure rearrangements (Figure 1, Table 2). Tobamovirus CP can bind tobacco chloroplast Ferredoxin I (Fd I) (Sun et al., 2013, Table 1), while TMV infection reduces the protein level of Fd I in tobacco leaves (Ma et al., 2008). Silencing of *Fd1* in tobacco plants leads to symptomatic chlorosis phenotype and enhances CP accumulation in chloroplast as well as virus multiplication, suggesting that the CP-Fd I interaction may contribute to the development of chlorosis and mosaic symptoms.

PVX CP and viral particles can also be detected in chloroplast of the infected plants, causing structural alteration of chloroplast membranes and grana stacks (Kozar and Sheludko, 1969; Qiao et al., 2009). PVX CP interacts with the chloroplast TP of plastocyanin (Table 1), and silencing of plastocyanin in *N. benthamiana* reduces viral symptom severity. In plastocyanin silenced plants, the accumulation of CP in chloroplasts was also reduced although total CP amount in infected cells did not change (Qiao et al., 2009), suggesting that the CP-plastocyanin interaction positively contributes to viral symptom-associated chloroplast abnormality (Figure 1, Table 2).

PVY CP is preferentially associated with the thylakoid membranes (Gunasinghe and Berger, 1991). PVY CP interacts with the large subunits of RuBisCO (RbCL) (Table 1) and this interaction may be involved in the production of mosaic and chlorosis symptoms (Feki et al., 2005). Further research indicates that chloroplast-targeted, but not cytosol-localized CP induces virus-like symptom (Naderi and Berger, 1997a,b). These observations suggest an intimate relationship between chloroplasts and PVY CP during the process of inhibiting PS II in viral pathogenesis.

CMV infection causes symptoms associated with chloroplast ultrastructure changes (Roberts and Wood, 1982; Shintaku et al., 1992; Mazidah et al., 2012). CMV CP can be transported into intact chloroplast promptly in a ATP-independent mode and the amount of CP into chloroplast correlated with the severity of mosaic symptoms (Liang et al., 1998). The single amino acid substitution at residue 129 in CP of CMV pepo strain is found to induce chloroplast abnormalities (Figure 1, Table 2) associated with the alteration of chlorosis severity (Shintaku et al., 1992; Suzuki et al., 1995; Mochizuki and Ohki, 2011; Mochizuki et al., 2014b), suggesting that CMV CP alone possess the virulence to induce chlorosis and chloroplast abnormalities in CMV-infected tobacco plants (Mochizuki and Ohki, 2011; Mochizuki et al., 2014b).

Viral CPs could also impose virulent effects from outside of the chloroplasts. A series of CP deletion mutants of TMV (Lindbeck et al., 1991) and ToMV spontaneous mutant ToMV-L_11_Y (Ohnishi et al., 2009) causes severe chlorosis associated with severe deformation and disruption of chloroplasts and the mutant CPs are shown to contribute to this severe chlorosis (Lindbeck et al., 1991; Ohnishi et al., 2009). Because the mutant CPs aggregate outside of chloroplasts, they may subvert the chloroplast development and cause the degradation of chloroplasts by interfering with the synthesis and transport of CPRPs (Lindbeck et al., 1991, 1992; Ohnishi et al., 2009).

Besides CPs, other viral components are also able to cause chloroplast malformation and contribute to symptom. For example, transgenic expression of CaMV transactivator/viroplasmin (Tav) protein in tobacco plants results in a virus-like chlorosis symptom associated with the abnormal thylakoid stacks (Figure 1, Table 2) and reduces expression of CPRGs (Waliullah et al., 2014). The potexvirus AltMV TGB3, different from its counterpart PVX TGB3, has a chloroplast-targeting signal and preferentially accumulates around the chloroplast membrane (Lim et al., 2010). Overexpression of AltMV TGB3 causes vesiculation at the chloroplast membrane (Figure 1, Table 2) and veinal necrosis symptom (Lim et al., 2010; Jang et al., 2013). AltMV TGB3 strongly interacts with PS II oxygen-evolving complex protein PsbO and this interaction is believed to have a crucial role in viral symptom development and chloroplast disruption (Jang et al., 2013). In PVY-infected cells, viral multifunctional protein HC-Pro may contribute to the change in the number and size of chloroplast by interfering with the normal activity of the chloroplast division-related factor MinD through direct protein interaction (Jin et al., 2007, Table 1). The tenuivirus RSV NSvc4 protein functions as an intercellular movement protein and is localized to PD as well as chloroplast in infected cells. Over-expression of NSvc4 exacerbated malformations of chloroplast (Figure 1, Table 2) and disease symptoms. Interestingly, the chloroplast localization of NSvc4 is dispensable for the symptom determination while the NSvc4 transmembrane domain probably affects the chloroplast from outside (Xu and Zhou, 2012).

### Effect of virus infection on expression of chloroplast-targeted proteins

Studies on the effect of virus infection on expression of chloroplast proteins at the transcriptomic and proteomic levels provide insights into the molecular events during symptom expression. In the susceptible plant response to virus infection, the majority of significantly changed proteins are identified to be located in chloroplasts or associated with chloroplast membranes. Most of them are down-regulated and correlate with the severity of chlorosis (Dardick, 2007; Shimizu et al., 2007; Lu et al., 2012; Rodríguez et al., 2012; Kundu et al., 2013; Wu et al., 2013; Mochizuki et al., 2014a). During virus infection, CPRPs represent the most common viral targets. Among them, the light harvesting antenna complex (Naidu et al., 1984a,b, 1986; Liu et al., 2014) and the oxygen evolving complex (OEC) (Takahashi et al., 1991; Takahashi and Ehara, 1992; Pérez-Bueno et al., 2004; Sui et al., 2006; Wang et al., 2015) of PS II are in thylakoid, while RbCS and RubisCO activase (RCA, an AAA-ATPase family protein) are in chloroplast stroma (Díaz-Vivancos et al., 2008; Pineda et al., 2010; Moshe et al., 2012; Kundu et al., 2013).

As the biosynthesis of CPRPs is a complicated process with a series of steps (Seidler, 1996), plant virus can affect CPRPs at varied levels including transcription, post-transcription, translation, transportation into the chloroplast, assembly and degradation in chloroplast, to contribute to symptom development (Lehto et al., 2003; Pérez-Bueno et al., 2004).

Several plant viruses perturb CPRPs expression at transcription level either in chloroplast or via retrograde signaling into nucleus. Infection of TMV *flavum* strain leads to a total depletion of PS II core complex and OEC, including chloroplast-encoded CPRP PsbA and nuclear-encoded CPRPs LhcB1, LhcB2 (light-harvesting chlorophyll a/b-binding protein B1, B2) and PsbO. However, the *PsbA* mRNA accumulated to a higher level in the infected leaves (Lehto et al., 2003). Thus, TMV *flavum* may block PsbA translation via reducing the level of chloroplast ribosomal RNA (Fraser, 1969) and inhibit the transcription of nuclear-encoded CPRGs through feed-back signaling (Lehto et al., 2003). Similarly, in the case of CMV pepo strain and its CP_129_ mutant isolates, the down-regulation patterns of transcription levels of different CPRGs correlated with the amino acid substitution in the CP protein of the relative isolates, where CMV CP probably repress the transcription of CPRGs via the retrograde signaling from chloroplast into nucleus (Mochizuki et al., 2014a).

It is interesting that plant virus can also exploit host RNA silencing machinery to manipulate CPRGs at post-transcription level. The enlightening evidence is illustrated by CMV-Y satellite (CMV-Y-sat) RNA which can disturb chloroplast function and induce disease symptoms (Shimura et al., 2011; Smith et al., 2011). A 22-nt siRNA derived from CMV-Y-sat RNA targets the *magnesium protoporphyrin chelatase subunit I* (*ChlI*) gene transcripts and down-regulates its expression by RNA silencing (Table 1), which leads to a more sever symptom characterized as bright yellow mosaic (Takanami, 1981; Shimura et al., 2011; Smith et al., 2011). In addition, infection by viroids (small non-protein-coding RNAs) results in the production of viroid-derived small RNAs (vd-sRNAs) (Papaefthimiou et al., 2001; Martínez de Alba et al., 2002). Peach latent mosaic viroid (PLMVd) belongs to family *Avsunviroidae* whose members replicate in chloroplast, and may elicit an albino-variegated phenotype (peach calico, PC) with blocked chloroplast development and depletion of chloroplast-encoded proteins (Rodio et al., 2007). The PLMVd variants associated with PC contain an insertion of 12–14 nt that folds into a hairpin with a U-rich tetraloop, the sequence of which is critical for inciting the albino phenotype. Actually, vd-sRNAs from the hairpin insertion induce cleavage of the mRNA encoding the CPRP chloroplastic heat-shock protein 90 (cHSP90) as predicted by RNA silencing, eventually resulting in PC symptoms (Navarro et al., 2012).

In addition to the virus-derived small RNAs, plant viruses may also modify host microRNA (miRNA) pathway for targeting CPRGs transcripts. The tenuivirus RSV, causing a devastating disease in East Asia countries, hijacks CPRP during infection and perturbs photosynthesis (Satoh et al., 2010; Shi et al., 2016). The perturbation of photosynthesis by RSV is probably caused by up-regulating a special miRNA that targets key genes in chloroplast zeaxanthin cycle, which impairs chloroplast structure and function (Yang et al., 2016).

Viral factors may reduce the level of CPRPs by direct association with target proteins. Tobamoviruses CPs particularly associate with the PS II complex and reduce the levels of PsbP and PsbQ (Hodgson et al., 1989; Pérez-Bueno et al., 2004; Sui et al., 2006). PVY HC-Pro can reduce the amount of ATP synthase complex by interaction with the NtCF1β-subunit in both the PVY-infected (Table 1) and the HC-Pro transgenic tobacco plants, leading to a decreased photosynthetic rate (Tu et al., 2015). Potyviruses TuMV, SMV, SYSV, and OYDV may hijack RbCS and/or RbCL via the interaction with P3 or P3N-PIPO during infection to perturb photosynthetic activity (Lin et al., 2011). Potyvirus SCMV infection significantly down-regulates mRNA level of photosynthetic Fd V rather than that of the other isoproteins (Fd I and Fd II) in maize, while SCMV HC-Pro specifically interacts with the chloroplast precursor of Fd V via TP in cytoplasm outside the chloroplasts (Table 1), suggesting that SCMV HC-Pro perturbs the importing of Fd V into chloroplasts and leads to structure and function disturbance of chloroplast (Cheng et al., 2008). Potyvirus SMV P1 (a serine protease) strongly interacts with host plant-derived, but only weakly with non-host *Arabidopsis*-derived, Rieske Fe/S protein of cytochrome b6/f complex, an indispensable component of the photosynthetic electron transport chain in chloroplasts (Table 1), suggesting that SMV P1-Rieske Fe/S protein interaction is involved in symptom development (Shi et al., 2007). RSV disease specific protein (SP) is a symptom determinant protein and its overexpression enhances RSV symptom (Kong et al., 2014). During RSV infection, accumulation of SP is associated with alteration in structure and function of chloroplast. SP interacts with 23-kD OEC PsbP, and relocates PsbP from chloroplast into cytoplasm (Table 1), while silencing of *PsbP* enhances disease symptom severity and virus accumulation (Kong et al., 2014).

## Chloroplast is involved in the process of the plant virus life cycle

Increasing studies have unraveled that chloroplast constituents participate in different stages during virus infection. For example, chloroplast is reported to be associated with viral uncoating, an important step of replication (Xiang et al., 2006). Tombusvirus CNV CP harbors an arm region of 38 amino acids that functions as a chloroplast TP to direct CP import to the chloroplast stroma, which is critical for viral disassembly. CNV CP mutant deficient in exposure of the arm region is inefficient to establish infection, highlighting the crucial role of chloroplast targeting in CNV uncoating (Xiang et al., 2006).

### Chloroplast and its factors participate in virus replication

Chloroplast affords compartment and membrane contents for the replication of plant viruses and probably helps them to evade the RNA-mediated defense response (Ahlquist et al., 2003; Dreher, 2004; Torrance et al., 2006). Plant viruses propagate via RNA-protein complex named viral replication complexes (VRCs), which are the factory for producing progeny viruses (Más and Beachy, 1998, 2000; Asurmendi et al., 2004). During replication of RNA viruses, double-strand RNA (dsRNA) is generated as an intermediate product. As a response against virus infection, the dsRNA replication intermediates can be detected by the host RNA silencing machinery (Angell and Baulcombe, 1997; Baulcombe, 1999). Correspondingly, plant viruses have evolved some mechanisms by encoding viral suppressor of RNA silencing or by associating replication with host membranes (Ahlquist, 2002; Ahlquist et al., 2003). For a large group of viruses, VRCs are associated with the chloroplast envelope, particularly the peripheral vesicles and cytoplasmic invaginations in chloroplast (Figure 1, Table 2), including alfamovirus AMV (de Graaff et al., 1993), hordeivirus BSMV (Carroll, 1970; Torrance et al., 2006), potyviruses MDMV (Mayhew and Ford, 1974), PPV (Martin et al., 1995), TEV (Gadh and Hari, 1986), TuMV (Kitajima and Costa, 1973), and tymovirus TYMV (Lafleche et al., 1972; Bové and Bové, 1985; Garnier et al., 1986; Lesemann, 1991; Dreher, 2004). The chloroplast membrane associated organization probably helps to shield viral RNAs from recognition by host RNA silencing machinery (Dreher, 2004).

Viral factors, either viral genomic RNAs or proteins, can mediate the chloroplast targeting of VRCs for replication and subsequent virion assembly (Prod'homme et al., 2003; Jakubiec et al., 2004; Torrance et al., 2006). BSMV replicative dsRNA intermediates exist in the chloroplast peripheral vesicles during infection (McMullen et al., 1978; Lin and Langenberg, 1984, 1985; Torrance et al., 2006); in the presence of the viral genome RNA, both TGB2 and γb can be recruited to chloroplasts for virus replication (Torrance et al., 2006). The low pH condition of chloroplast vesicles where TYMV RNA is synthesized is required for the interaction between viral RNA and CP to process virion assembly (Rohozinski and Hancock, 1996). The TYMV VRC-associated membrane vesicles localize at the chloroplast envelope (Prod'homme et al., 2001). TYMV N-terminal replication protein (140 K) is a key organizer of TYMV VRCs assembly and a major determinant for chloroplast localization of TYMV for replication. The 140 K protein can localize to the chloroplast envelope autonomously and interacts with the C-terminal replication protein (66 K) to mediate the targeting of 66 K to the chloroplast envelope (Prod'homme et al., 2003; Jakubiec et al., 2004). TuMV 6 K protein (6 K or 6 K2) can autonomously allocate to chloroplast membrane and promote the adhesion of the adjacent chloroplasts via actomyosin motility system in infected host cells. During the infection, TuMV 6 K induces the formation of 6 K-containing membranous vesicles at endoplasmic reticulum exit sites and sequentially traffic to chloroplast, while the chloroplast-bounded 6 K-vesicles are recruited to VRCs containing viral dsRNA (Wei et al., 2010), supporting the idea that the chloroplast-bound 6 K vesicles are the cellular compartment for TuMV replication. Blocking the fusion of virus-induced vesicles with chloroplasts by the inhibition of SNARE protein Syp71 significantly reduced the viral infection (Wei et al., 2013).

Special chloroplast components are involved in the targeting of VRCs to chloroplast. The lipid in chloroplast membrane can associate with pomovirus PMTV TGB2 (Table 1) and facilitate the viral RNA to localize to chloroplast membranes for replication (Cowan et al., 2012). Furthermore, chloroplast factors also participate in the formation of VRCs. Proteomic analysis suggests that sobemovirus RYMV recruits CPRPs such as Ferredoxin-NADP reductase (FNR), RbCS, RCA, and chaperonin 60 to its VRCs during all the infectious stages including replication, long-distance trafficking and symptoms development (Brizard et al., 2006). The 43 kD CPRP chloroplast phosphoglycerate kinase (cPGK) specifically interacts with 3′-UTR of the potexvirus BaMV genomic RNA (Lin et al., 2007, Table 1). Silencing of *Nb*-*cPGK* or mislocalization of cPGK protein reduced BaMV accumulation, suggesting that cPGK may mediate BaMV RNA targeting to chloroplast for replication (Cheng et al., 2013). Interestingly, in *Arabidopsis* genotype Cvi-0 the natural recessive resistance gene *rwm1* against potyvirus WMV encodes a mutated version of cPGK (Ouibrahim et al., 2014), illuminating that the conserved CPRP cPGK may be required for successful replication and infection of a range of plant viruses (Lin et al., 2007; Ouibrahim et al., 2014).

### Chloroplast factors participate in viral movement

The intercellular trafficking and systemic spreading of plant virus need movement proteins (MPs) to fulfill the transport via symplastic routes within plant hosts (Wolf et al., 1989; Ding et al., 1992; Imlau et al., 1999; Lazarowitz and Beachy, 1999). To facilitate virus movement, varied MPs possess common features such as nucleic acid binding activity (Citovsky et al., 1990), specific plasmodesmata (PD) localization (Ding et al., 1992; Fujiwara et al., 1993) and the ability to increase the size exclusion limit of PD (Wolf et al., 1989).

Chloroplast and its factors also participate in virus movement. AltMV TGB3 has a chloroplast-targeted signal and accumulates preferentially in mesophyll cells, which is essential for virus movement. Mutation of the chloroplast-targeted signal in AltMV TGB3 impairs virus movement from epidermal into the mesophyll cells as well as viral long-distance traffic (Lim et al., 2010). Geminivirus AbMV MP interacts with chloroplast-targeted 70-kD heat shock protein (cpHSC70-1) and co-localized to chloroplasts (Table 1). Silencing of *cpHSC70*-1 affects chloroplast stability and causes a substantial reduction of AbMV movement but has no effect on viral DNA accumulation (Krenz et al., 2010, 2012). AbMV can replicate in chloroplast (Gröning et al., 1987, 1990) and induce the biogenesis of stromule network (Figure 1, Table 2). AbMV may use cpHSC70-1 for trafficking along chloroplast stromules into a neighboring cell or from plastids into the nucleus (Krenz et al., 2012).

Viral factors can interact with and hijack chloroplast factors from their normal function and to help viral movement. The CaMV multifunctional P6 protein is the most abundant present in VRCs (Hohn et al., 1997) and associates with PD (Rodriguez et al., 2014). Interestingly, CaMV P6 also interacts with the chloroplast unusual positioning protein1 (CHUP1) (Table 1) that is a thylakoid membrane-associated protein for mediating the routine movement of chloroplast on microfilaments in response to light intensity (Oikawa et al., 2003, 2008). Silencing of *CHUP1* slows the formation rate of CaMV local lesion (Angel et al., 2013). Thus, the CaMV P6 protein may mediate the intracellular movement of VRCs to the PD by binding to CHUP1 (Angel et al., 2013). Tobamoviruses ToMV and TMV MPs bind RbCS (Table 1) and the interaction occurs at PD (Zhao et al., 2013). Silencing of *RbCS* reduced intercellular movement and systemic trafficking of TMV and ToMV (Zhao et al., 2013). Thus, it may be a common strategy for tobamoviruses to hijack RbCS for efficient movement. In addition to MPs, tobamoviruses need their CPs for efficient long distance movement (Wisniewski et al., 1990; Reimann-Philipp and Beachy, 1993; Ryabov et al., 1999). ToMV CP-interacting protein-L (IP-L) is a chloroplast protein (Table 1) and is positively induced by ToMV infection (Zhang et al., 2008). Depletion of *IP-L* delayed ToMV systemic movement and symptoms (Li et al., 2005). Dianthovirus RCNMV MP interacts with chloroplast protein glyceraldehyde 3-phosphate dehydrogenase subunit A (GAPDH-A) (Table 1), while silencing of *GAPDH-A* inhibits viral MP localization to the cortical VRCs and reduces RCNMV multiplication in the inoculated leaves (Kaido et al., 2014). Therefore, GAPDH-A is relocated from chloroplast to cortical VRCs to facilitate viral cell-to-cell movement during RCNMV infection.

Based on the current studies, it is clear that plant viruses have evolved to utilize abundant chloroplast proteins to regulate their movement.

## Chloroplasts affect plant defense against viruses

**Figure 2:**
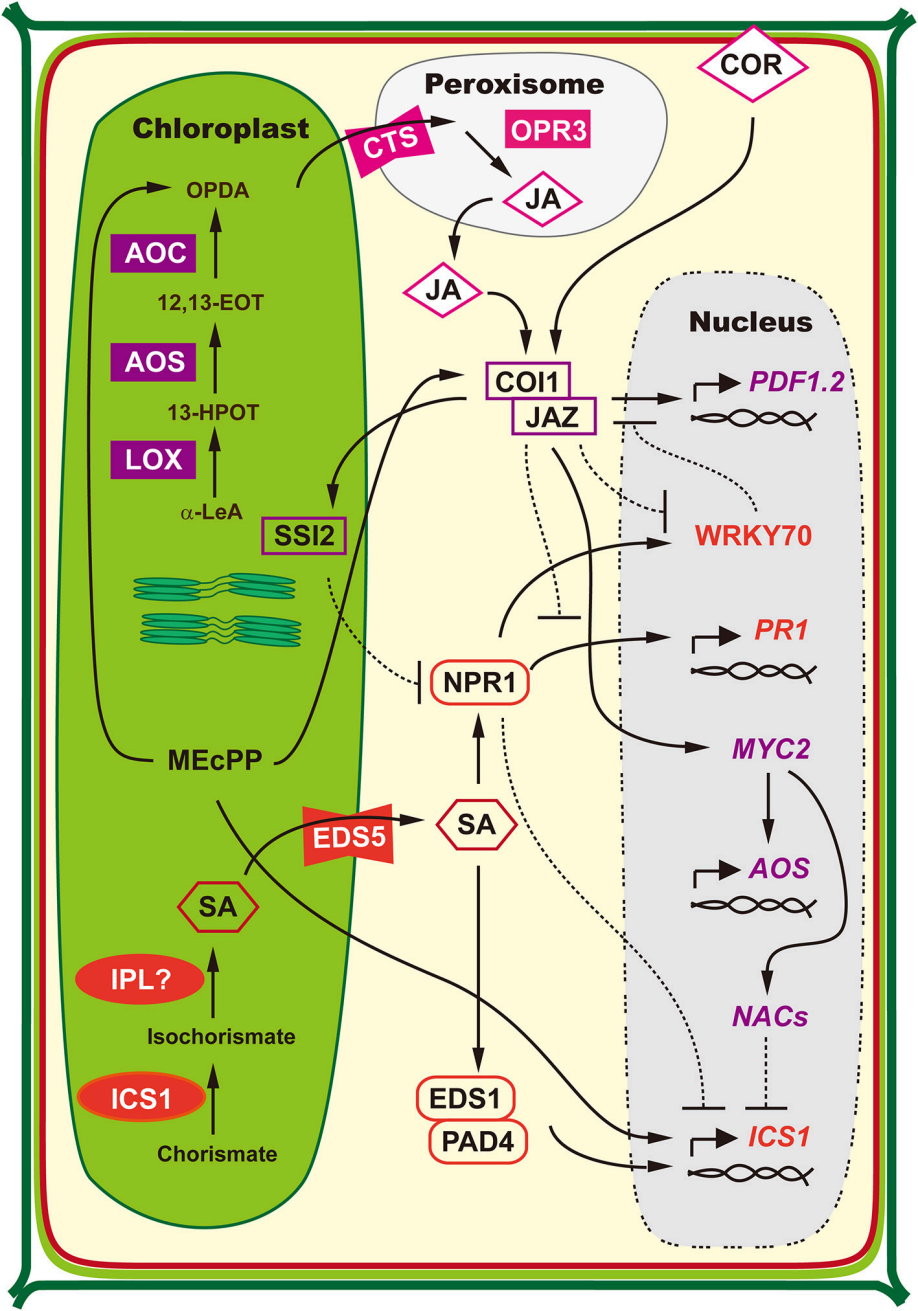
**Regulation of SA and JA Biosynthesis is Associated with Chloroplast**. SA biosynthesis is predominantly accomplished by nucleus-encoded chloroplast-located isochorismate synthase (ICS1). In chloroplasts, ICS catalyzes the conversion of chorismate into isochorismate, which is further converted to SA by undetermined isochorismate pyruvate lyase (IPL). The MATE-transporter ENHANCED DISEASE SUSCEPTIBILITY 5 (EDS5) is responsible for SA transportation from chloroplast into cytosol. Defense-elicited ENHANCED DISEASE SUSCEPTIBILITY 1 (EDS1) and PHYTOALEXIN DEFICIENT 4 (PAD4) complex works in a positive feedback loop to control SA synthesis, which is regulated by SA. While in a negative feedback loop, accumulation of ICS1-produced SA results in the deoligomerization of NON-EXPRESSOR OF PATHOGENESIS-RELATED GENES 1 (NPR1), which is then translocated into nucleus where it suppresses the *ICS1* expression (modified from Boatwright and Pajerowska-Mukhtar, 2013; Seyfferth and Tsuda, 2014). JA biosynthesis originates from polyunsaturated fatty acids released from chloroplast membranes. Firstly, α-linolenic acid (18:3) (α-LeA) is catalyzed by lipoxygenase (LOX) to yield the 13-hydroperoxy derivative 13(S)-hydroperoxy-octadecatrienoic acid (13-HPOT). The dehydration of 13-HPOT by allene oxide synthase (AOS) results in the formation of unstable 12, 13(S)-epoxy-octadecatrienoic acid (12,13-EOT), which is the committed step of JA biosynthesis. Then the 12,13-EOT is converted to 12-oxophytodienoic acid (OPDA) by allene oxide cyclase (AOC) through cyclization and concludes the chloroplast-localized part of JA biosynthesis. Subsequently, OPDA is released from chloroplasts and taken up into peroxisomes by transporter COMATOSE (CTS3). The remaining steps are located in peroxisomes and JA is generated through reduction of the cyclopentenone by OPDA reductase 3 (OPR3) and subsequent three cycles of β-oxidation for side-chain shortening. The JA co-receptor complex of CORONATINE INSENSITIVE1 (COI1) and the negative regulator JAZMONATE ZIM DOMAIN (JAZ) proteins regulates the positive feedback loop of JA biosynthesis. Formation of JA subjects JAZ to proteasomal degradation, which allows MYC2 to activate the JA biosynthesis genes such as AOS, AOC, and LOX (modified from Wasternack, 2007; Schaller and Stintzi, 2009; Wasternack and Hause, 2013). NPR1 is the central transcriptional regulator of SA-mediated defense responses and directly regulates *PATHOGENESIS-RELATED 1* (*PR1*) expression (Wang et al., 2006). By wounding or JA treatment, COI1–JAZ co-receptor promotes the degradation of JAZ and release the positively acting transcription factors that binds to JA-responsive promoters to initiate the transcription of JA-responsive genes, such as *PLANT DEFENSIN1.2* (*PDF1.2*) (Chini et al., 2007; Thines et al., 2007; Yan et al., 2009). During the antagonistic interplay between SA and JA, NPR1 suppresses COI1-JAZ mediated induction of JA-responsive genes via WRKY transcription factors, while JA also represses WRKY in COI1-dependent pathway (Li et al., 2004; Gao et al., 2011). On the other hand, the JA signaling proteins, such as chloroplast factor SUPPRESSOR OF SA INSENSITIVITY 2 (SSI2), negatively regulate SA-mediated NPR1-dependent defense responses (Kunkel and Brooks, 2002). Further, the phytotoxin coronatine (COR), a molecular mimic of JA, activates NAC transcription factors via COI1-JAZ and MYC2, which eventually inhibits SA accumulation through repressing *ICS1* expression (Zheng et al., 2012). In addition, the stress-induced methylerythritol cyclodiphosphate (MEcPP) acts as a plastid-to-nucleus retrograde signal to increase the transcription level of *ICS1* (Xiao et al., 2012). Meanwhile, MEcPP increase the level of JA precursor OPDA and induce JA-responsive genes via a COI1-dependent manner in the presence of high SA (Lemos et al., 2016). Solid lines with arrow head represent activation or promotion, dotted lines with bar head to represent deactivation or inhibition.

Several hormones regulate plant defense to viruses (Alazem and Lin, 2015). Two of them are salicylic acid (SA) and jasmonic acid (JA). Chloroplast is the crucial site for the biosynthesis of SA (Boatwright and Pajerowska-Mukhtar, 2013; Seyfferth and Tsuda, 2014) and JA (Wasternack, 2007; Schaller and Stintzi, 2009; Wasternack and Hause, 2013). Moreover, chloroplast factors are also involved in the regulation of antagonistic interactions of SA-JA synthesis and signaling (Kunkel and Brooks, 2002; Xiao et al., 2012; Zheng et al., 2012; Lemos et al., 2016). The chloroplast-related regulation of SA and JA biosynthesis is schemed in Figure 2.

SA is a small phenolic compound that plays central roles in plant defense against biotrophic pathogens and is essential for the establishment of local and systemic acquired resistance. The majority of pathogen-induced SA is synthesized via the isochorismate pathway in chloroplasts (Boatwright and Pajerowska-Mukhtar, 2013; Seyfferth and Tsuda, 2014). As a key activator of plant defense response, SA biosynthesis and signaling are activated during incompatible plant-virus interaction (Wildermuth et al., 2001; Garcion et al., 2008). Disruption of SA pathway compromises plant resistance against viruses (Alazem and Lin, 2015). In contrast, the application of SA or its analogs often delays the onset of viral infection and disease establishment by improving plant basal immunity (Radwan et al., 2006, 2007, 2008; Falcioni et al., 2014). A chloroplast-localized protein, named calcium-sensing receptor, is found to act upstream of SA accumulation to link chloroplasts to cytoplasmic-nuclear immune responses (Nomura et al., 2012).

JA is an oxylipin, or oxygenated fatty acid and is synthesized from linolenic acid by the octadecanoid pathway, whose biosynthesis starts with the conversion of linolenic acid to 12-oxo-phytodienoic acid (OPDA) in the chloroplast membranes (Turner et al., 2002). JA is thought to play a positive defense role in compatible plant-virus interactions (Alazem and Lin, 2015). For example, silencing of *Coronatine insensitive 1* (*COI1*), a gene involved in the JA signaling pathway, accelerates the development of symptoms caused by co-infection of PVX and PVY, and accumulation of viral titers at early stages of infection (García-Marcos et al., 2013).

The chloroplasts are major sites of the production of reactive oxygen species (ROS), and the photosynthetic electron transport chain is responsible for ROS generation (Asada, 2006; Muhlenbock et al., 2008). Superoxide anion (${\text{O}}_{2}^{-}$) is the primary reduced product of O_2_ photoreduction and its disproportionation produces H_2_O_2_ in chloroplast thylakoids (Asada, 2006; Muhlenbock et al., 2008). The burst of intracellular ROS can be detected during virus infection in both incompatible and compatible interactions (Allan et al., 2001; Hakmaoui et al., 2012). Chloroplast-sourced ROS are essential for hypersensitive response (HR) induced by incompatible defensive response (Torres et al., 2006; Zurbriggen et al., 2010).

The stromules could function to facilitate the magnification and transport of defensive signals into the nucleus. Interestingly, the stromules can be induced during *N*-mediated TMV resistance response. Further, a number of stromules surround nuclei during plant defense response, which is correlated with the accumulation of chloroplast-localized defense protein NRIP1 and H_2_O_2_ in the nucleus. In the absence of virus infection, suppression of chloroplast *CHUP1* induces stromules and enhances programmed cell death constitutively (Caplan et al., 2015; Gu and Dong, 2015). In addition, the ultrastructural changes in chloroplast can also be a part of resistant response. For examples, during the hypersensitive reaction of *N*-mediated TMV resistance, the chloroplasts swelled and the membrane burst before tonoplast ruptured (da Graça and Martin, 1975). During the course of lesion development caused by the nepovirus TRSV, the changes in chloroplast ultrastructure (rounding of chloroplasts) enlighten that chloroplast disturbance could reflect plant-virus incompatible responses (White and Sehgal, 1993). The ultrastructure aberrations of chloroplast represent the intensity of apoptotic processes in PVY^NTN^ infection (Pompe-Novak et al., 2001). Thus, the malformation of chloroplast may also indicate a defense response in compatible host-virus interaction.

Removal of the lower epidermis from cowpea and tobacco leaves inoculated with TMV or TNV resulted in reduction of local lesion numbers, indicating that the chloroplast-free epidermal cells possess an active role in virus infection (Wieringabrants, 1981). Further, chloroplast may also have a role in host defense against virus during the compatible plant-virus interaction. Previous studies found that light could influence host susceptibility to virus infection. Despite there is a report that a short burst of light after dark treatment enhances plant susceptibility to TMV infection (Helms and McIntyre, 1967), in most cases, low light and dark treatment is beneficial for viruses to establish infection and increase host's susceptibility compared to light treatment (Bawden and Roberts, 1947; Matthews, 1953; Wiltshire, 1956; Helms, 1965; Helms and McIntyre, 1967; Cheo, 1971; Manfre et al., 2011). The negative correlation between light and infectivity suggest that the robust photosynthesis and chloroplast function play a positive role in defense response during plant-virus interactions.

In compatible plant-virus interactions, some chloroplast factors are sequestrated by virus to block antiviral defense and fuel virus infection. For examples, AMV CP is essential for virus replication and encapsidation, and interacts with the chloroplast protein PsbP in the cytosol (Table 1), while mutations that prevent the dimerization of CP abolish this interaction (Balasubramaniam et al., 2014). Interestingly, overexpression of *PsbP* markedly reduced AMV replication in infected leaves, suggesting that there is a potential PsbP-mediated antiviral mechanism which was sequestered by CP-PsbP interaction (Balasubramaniam et al., 2014).

TMV 126-kD replicase associates with several CPRPs (Table 1) such as PsbO (Abbink et al., 2002), RCA and ATP-synthase γ-subunit (AtpC) (Bhat et al., 2013). Silencing of *PsbO* results in leaf chlorosis and elevated replication of several viruses including TMV, AMV, and PVX (Abbink et al., 2002). Similarly, suppression of *AtpC* and *RCA* enhances the accumulation of TMV and TVCV (Bhat et al., 2013). In addition, TMV infection specifically decreased the expression levels of *AtpC, RCA*, and *PsbO* (Abbink et al., 2002; Bhat et al., 2013). Further, silencing of *RbCS* enhances host susceptibility to ToMV and TMV, which is be accompanied by the reduced expression of pathogen related gene *PR-1a* (Zhao et al., 2013). These findings suggest that these CPRPs (RbCS, AtpC, RCA, and PsbO) play roles in plant defense against TMV, and TMV has evolved a strategy to suppress the defense of host plants for optimizing their own propagation.

The cylindrical inclusion (CI) protein of potyviruses is required for virus replication and cell-to-cell movement. CI protein from PPV and TVMV interacts with photosystem I PSI-K protein (Table 1), the product of the gene *psaK* in yeast (Jimenez et al., 2006). Overexpression of PPV CI reduces protein level of PSI-K while silencing or knockout of *psaK* enhances PPV accumulation in *N. benthamiana* and *Arabidopsis*, suggesting that chloroplast-localized PSI-K protein could have an antiviral role (Jimenez et al., 2006).

AltMV TGB1 can bind several chloroplast factors (Table 1), such as light harvesting chlorophyll-protein complex I subunit A4 (LhcA4), chlorophyll a/b binding protein 1 (LHB1B2), chloroplast-localized IscA-like protein (CPISCA) and chloroplast β-ATPase (CF1β) (Seo et al., 2014). Among those chloroplast proteins, CF1β selectively binds the wild type TGB1_L88_ with high RNAi suppressor activity (Table 1) but not the natural variant TGB1_P88_ with reduced silencing suppressor activity (Seo et al., 2014). During infection with wild type AltMV, silencing of *CF1*β specifically causes severe necrosis without a significant change of viral RNAs, suggesting a direct role of *CF1*β responding to TGB1_L88_ to induce defense responses (Seo et al., 2014). Taken together, the above reports indicate that the chloroplast plays an important defense role during virus invasion.

During incompatible plant-virus interactions, some chloroplast factors also participate in plant defense against viruses. For examples, in TMV resistance gene *N* containing tobacco, N receptor interacting protein 1 (NRIP1), a rhodanese sulfurtransferase which is destined to chloroplast under normal conditions, associates with both the tobacco N receptor and 126 K replicase during TMV infection; its relocation from chloroplast to cytoplasm and nucleus is required for *N*-mediated resistance to TMV (Caplan et al., 2008). Moreover, depletion of *RbCS* compromises *Tm-2*^*2*^ mediated extreme resistance against ToMV and TMV (Zhao et al., 2013). In addition, chloroplast-localized calcium-sensing receptor is found to be involved in stromal Ca^2+^ transients and responsible for both basal resistance and *R* gene-mediated defense (Nomura et al., 2012). These observations are consistent with the idea that chloroplasts have a critical role in plant immunity as a major site for the production for ROS, SA, and JA, important mediators of plant immunity.

Taken together, chloroplast factors participate in both basal defense and *R* gene mediated immunity against viruses.

## Conclusions and future perspectives

The disturbance of chloroplast structure or components is often involved in symptom development and some chloroplast proteins help viruses to fulfill their infection cycle in plants. On the other hand, chloroplast factors seem to play active roles in plant defense against viruses. This is consistent with the idea that ROS, SA, and JA are produced in chloroplast (Heiber et al., 2014).

So far, some chloroplast factors involved in virus symptomology, infection cycle or antiviral defense have been identified, and their roles in virus infection have been characterized. Some findings can explain phenomena observed in early reports. However, our understanding about chloroplast-virus interaction is still quite poor. In the future, we need to identify more chloroplast factors that take part in virus infection and plant defense against viruses, to unravel their precise role and functional mechanism during plant-virus interactions, to investigate how viruses modulate expression of *CPRGs* and chloroplast-derived signaling to affect plant response to viruses, and how viral factors or defense signals traffic between chloroplast and other cellular compartments. Further progress in understanding of chloroplast-virus interactions will open new possibilities in controlling virus infection by regulating host factor's expression level.

## Author contributions

JZ wrote most part of this manuscript. XZ helped to write this manuscript. YL, YH supervised, revised and complemented the writing.

## Funding

This work was supported by the National Natural Science Foundation of China (31530059, 31470254, 31300134, 31270182, and 31370180), the National Basic Research Program of China (2014CB138400), the Special Fund for Agro-scientific Research in the Public Interest of China (201303028), and the China Postdoctoral Science Foundation (2014M550049), the Initial Funding of Zhejiang Academy of Agricultural Sciences, and the Cultural Funding for Youth Talent of Zhejiang Academy of Agricultural Sciences (2015R21R08E03).

### Conflict of interest statement

The authors declare that the research was conducted in the absence of any commercial or financial relationships that could be construed as a potential conflict of interest.

## Abbreviations

AbMV: Abutilon mosaic virus
AltMV: Alternanthera mosaic virus
AMV: Alfalfa mosaic virus
BaMV: Bamboo mosaic virus
BSMV: Barley stripe mosaic virus
CaMV: Cauliflower mosaic virus
CI protein: Cylindrical inclusion protein
CMV: Cucumber mosaic virus
CNV: Cucumber necrosis virus
CP: Coat protein, Capsid protein
CPRG/CPRP: chloroplast photosynthesis-related gene/protein
HC-Pro: Helper component protein proteinase
JA: Jasmonic acid
MDMV: Maize dwarf mosaic virus
MP: Movement protein
OEC: Oxygen evolving complex
OYDV: Onion yellow dwarf virus
PD: plasmodesmata
PMTV: Potato mop-top virus
PPV: Plum pox virus
PS II: photosystem II
PVX: Potato virus X
PVY: Potato virus Y
RCNMV: Red clover necrotic mosaic virus
RdRP: RNA-dependent RNA polymerase
*R* gene: *Resistance* gene
ROS: Reactive oxygen species
RSV: Rice stripe virus
RuBisCO: Ribulose-1,5-bisphosphate carboxylase/oxygenase
RYMV: Rice yellow mottle virus
SA: Salicylic acid
SCMV: Sugarcane mosaic virus
siRNA: small interfering RNA
SMV: Soybean mosaic virus
SNARE: soluble NSF attachment protein receptor
SYSV: Shallot yellow stripe virus
TBSV: Tomato bushy stunt virus
TEV: Tobacco etch virus
TGB proteins: Triple gene block proteins
TMV: Tobacco mosaic virus
TNV: Tobacco necrosis virus
ToMV: Tomato mosaic virus
TRSV: Tobacco ringspot virus
TuMV: Turnip mosaic virus
TVMV: Tobacco vein-mottling virus
TYMV: Turnip yellow mosaic virus
VRC: Viral replication complex
WMV: Watermelon mosaic virus.
